# Allergic rhinitis is associated with atmospheric SO_2_: Follow-up study of children from elementary schools in Ulsan, Korea

**DOI:** 10.1371/journal.pone.0248624

**Published:** 2021-03-18

**Authors:** Suk Hwan Kim, Jiho Lee, Inbo Oh, Yeonsuh Oh, Chang Sun Sim, Jin-Hee Bang, Jungsun Park, Yangho Kim

**Affiliations:** 1 POSCO Health Center, POSCO, Pohang, Republic of Korea; 2 Department of Occupational and Environmental Medicine, Ulsan University Hospital, University of Ulsan College of Medicine, Ulsan, Republic of Korea; 3 Environmental Health Center, University of Ulsan College of Medicine, Ulsan, Republic of Korea; 4 Department of Occupational Health, Catholic University of Daegu, Gyeongsan, Republic of Korea; The Ohio State University, UNITED STATES

## Abstract

**Objectives:**

The purpose of this study was to examine the association of allergic rhinitis with air pollutant concentrations using the follow-up data of elementary school children in Ulsan, Korea.

**Methods:**

All students of four elementary schools in Ulsan, South Korea were surveyed at two-year intervals. The survey used data collected five times, over a nine-year period from June 2009 to April 2018. The questionnaire used in the survey was a modified version of the ISAAC (International society of asthma and allergy of children) questionnaire. A skin prick test (SPT) was performed with 24 standard antigens. To estimate the levels of exposure to outdoor air pollution, the concentrations of sulfur dioxide (SO_2_), nitrogen dioxide (NO_2_), ozone (O_3_), carbon monoxide (CO), and particulate matter 10 μm or less in diameter (PM10) were used. As a dependent variable, a history of allergic rhinitis diagnosed by a doctor during the last 1-year was considered. Logistic regression analysis was used to select variables suitable for the statistical model. The selected variables were then used to assess their association with the dependent variable using the generalized estimation equation.

**Results:**

Among environmental factors, SO_2_ was associated with a high risk and PM10 was associated with a low risk of allergic rhinitis. The risk of allergic rhinitis from living in a house built within the last year was high, and the risk from living in a multi-family house or apartment was higher than that from living in a segregated house. History of allergic diseases in the family was a high-risk factor for allergic rhinitis. There was a relationship between a history of bronchiolitis at less than 2 years of age and a high risk of allergic rhinitis. Boys were at a higher risk than girls.

**Conclusion:**

From the follow-up data of elementary school students in Ulsan, Korea, the concentration of SO_2_, which is an indicator of the degree of industrialization, was related to the prevalence of allergic rhinitis. Among all the risk factors, history of allergic disease in the parents was the most important factor, and the study reconfirmed the results of the previous studies.

## Introduction

According to a report by the World Allergy Organization in 2013, approximately 20% of the global population has an allergic disease such as asthma, allergic rhinitis, atopic dermatitis, and allergic conjunctivitis [1]. A recent systematic review of the prevalence rates of allergic diseases in Korea over the past 30 years found continuous increases in the rates of allergic rhinitis, allergic conjunctivitis, atopic dermatitis, and food allergy, although there was no distinct trend in the prevalence of asthma [2]. A notable observation was that the prevalence of allergic rhinitis in children has continuously increased in recent decades [2–4].

Numerous previous studies on the causes of allergic diseases have focused on natural antigens such as house dust mites [5–7], dogs or cats [8, 9], and cockroaches [10], as well as on personal factors such as history of vaccination, use of antibiotics, and changes in lifestyle [11, 12]. A study has reported that the byproducts of diesel combustion might act as allergic sensitizers [13], while others have reported that people living in regions with severe air pollution due to industrialization showed lower sensitization to allergens [14]. The health effects of air pollutants remain unclear, as myriad chemical substances are developed and released each year. Thus, further studies on air pollutants are needed.

In the European Union guidelines on the testing and management of air quality, the following substances were set as targets to be controlled in consideration of their negative impacts on the human body and plants: O_3_, NO_2_, SO_2_, CO, and PM10 [15]. However, the results of studies on air pollutants vary significantly depending on the number of subjects, study design, target substance, measuring device, duration of measurement, region of measurement, and the methods [16, 17]. For example, one previous study examined the correlation between the concentration of SO_2_ and the risk of allergic rhinitis in elementary school students (odds ratio: 1.30) [18], while another study determined the correlation between SO_2_, CO, and NO_2_ concentrations and the prevalence of allergic rhinitis [19]. There is even a case in which the same study obtained inconsistent results depending on how subjects were grouped; specifically, an analysis of all subjects showed a correlation between NO_2_ concentration and increased prevalence of allergic rhinitis but an analysis of subgroups defined by the region of air pollution showed a correlation with reduced prevalence [20]. Most previous studies on this topic were cross-sectional, and had limitations related to study design. Thus, there is a need for a cohort study that analyzes patients over time.

Ulsan is a large metropolitan city located on the south-eastern coast of South Korea, with a population of approximately 1.1 million (as of 2019). Urban areas of Ulsan are close to national industrial complexes located along the labyrinthine coast, including petrochemical complex, car manufacturers, and shipbuilders [21]. According to the Annual Report of Air Quality in Korea 2017, the average level of SO_2_ in Ulsan is considerably higher than that in non-urban areas in South Korea and is notably higher than that in other metropolitan cities; for example, since 2000, in two years (2001 and 2004) Ulsan was the only city in South Korea in which SO_2_ levels exceeded the threshold level in the annual record (July 2017, 9 ppb) [22]. In addition, recent studies have shown a significant increase in the prevalence of allergic rhinitis in children in Ulsan [23]. There is growing concern in Ulsan about the effects of air pollutants on allergic rhinitis in areas with high air pollution due to the national industrial complexes.

Thus, in the present work we examined the association of allergic rhinitis with air pollutant concentrations, mainly SO_2_, using the follow-up data of elementary school children in Ulsan, Korea.

## Materials and methods

### Study design

**Fig 1:**
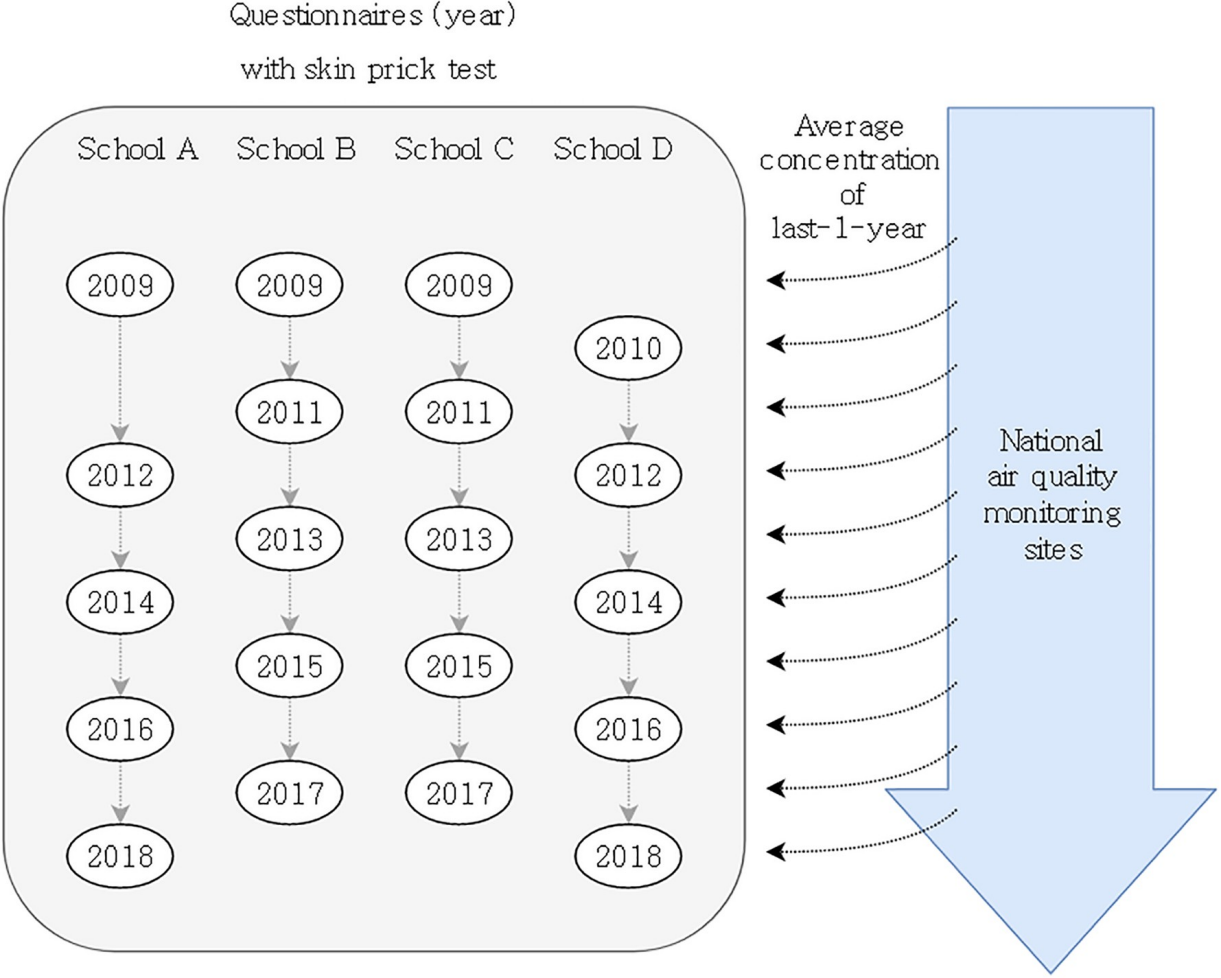
Study design. School A: Suburban residential, B: Near industrial area, C: Coastal residential, D: Central urban.

All data were from survey questionnaires, the results of the skin prick test (SPT, an objective indicator of allergic sensitization), and measurements of air pollutants (Fig 1). The overall study design is presented in Fig 1. Detailed description on Fig 1 were provided in subsections of ‘Study area’ and ‘Data collection’.

### Study area

The average temperature and wind speed in Ulsan were 14.1°C and 2.1 m/s, and the temperature is relatively higher than that in the other regions (1981–2010; the top 12%-tile and 38%-tile, respectively, among the 73 national monitoring sites managed by the Korea Meteorological Administration). In contrast, the average relative humidity was markedly lower at 64.2% (the low 7%-tile), which uniquely distinguishes this region from others [24]. North-westerly winds are predominant throughout the year, while the frequency of easterly and south-westerly winds increases during spring and summer.

O_3_ concentration in Ulsan has shown a rising trend as that in other cities [25]. In the case of volatile organic compounds, the level is higher in regions with industrial complexes than that in residential regions, clearly demonstrating the impact of the industrial activities in Ulsan [26]. Considering the environmental characteristics such as large-scale industrial facilities located in the coastal areas and the urban areas nearby regions, the surrounding mountains and frequent wind from the sea, it is likely that a large proportion of the urban population are frequently exposed to pollutants from the industrial complexes and city traffic [21].

**Fig 2:**
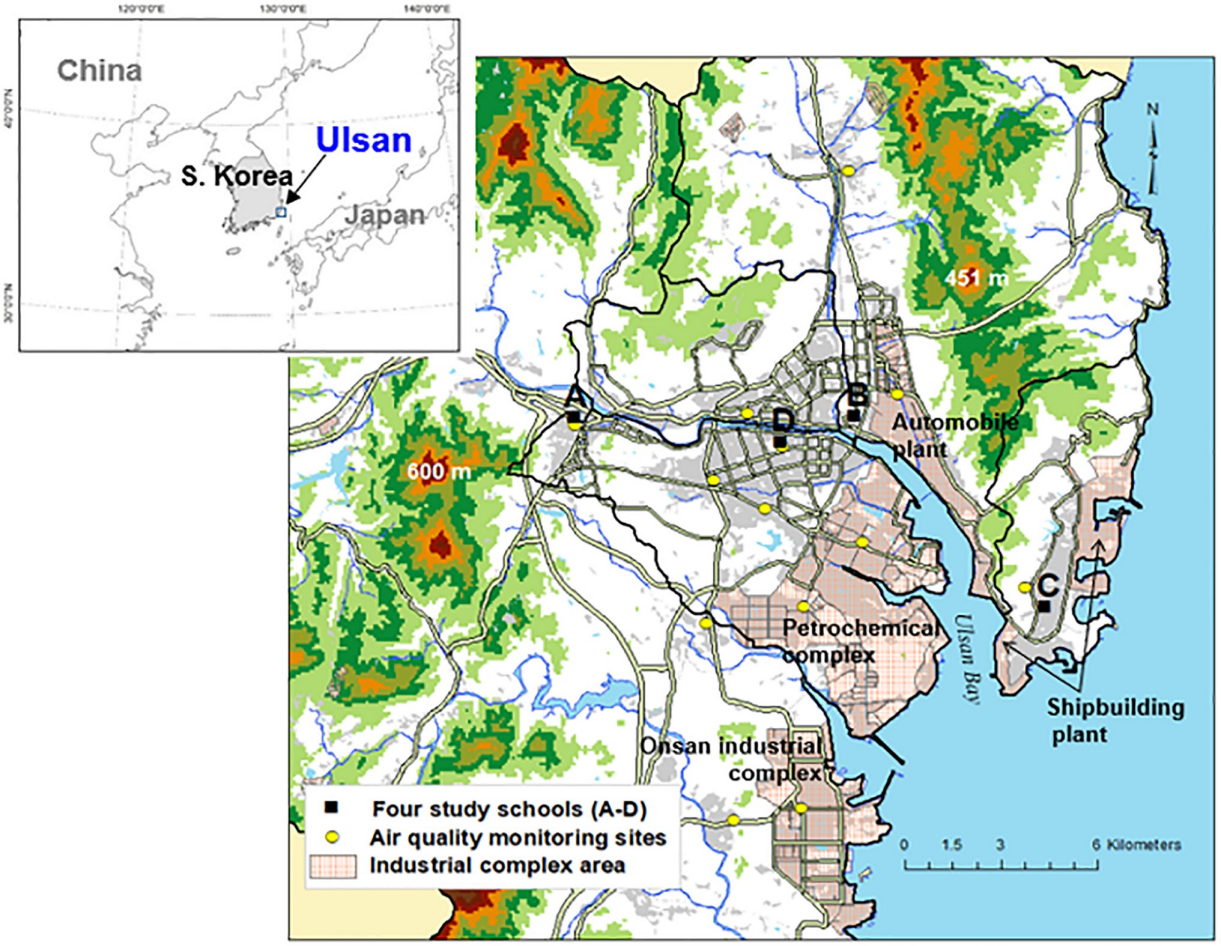
Geographical features in Ulsan and the location of the study schools (A-D) and air quality monitoring sites (AQMS). Light red shaded regions are industrial area. Gray shaded regions and thick lines indicate built-up areas and main roads, respectively.

Among the locations in the region of Ulsan that show varying atmospheric characteristics, four elementary schools were selected (Fig 2) [21]. The data for this study were collected from the students of all years at the four schools. The location of the first school (A) had the characteristics of a suburban, residential area, and was relatively far away from the city center and the industrial complex. The second school (B) was located close to the industrial complex with the probability of being under a significant influence of air pollutants released from the large industrial complex of the Ulsan region. A global car manufacturer was found across the 8-lane road, and across the river was the petrochemical complex. The location of the third school (C) had the characteristics of a coastal and residential area, as it was near the sea with a lower temperature and higher wind speed than in the urban area [27]. The fourth school (D) was in the central urban area with multipurpose buildings including shopping quarters and residential areas. Table 1 summarizes the characteristics of each elementary school.

**Table 1:** Characteristics of the four examined schools during 2019.

	Suburban residential (A)	Near industrial (B)	Coastal residential (C)	Central urban (D)
Students per grade	100	140	90	180
Year when built	2000	2003	2004	1991
Floors	6	6	5	5
Playground	+	+	+	+
Gymnasium	+	+	+	+

School A-D: see Fig 2.

While the four schools did not significantly vary in terms of the overall size or the presence of a playing field or an indoor gym, the elementary school in the central urban area was built before the other schools.

### Data collection

#### Survey questionnaire

The survey questionnaire was administered to all the students of all years at the four selected elementary schools. The survey has been conducted by the Environment Health Care Center of Ulsan University Hospital since June 2009 until 2019. Each year, the survey was conducted at two schools; thus, each school underwent the survey at two-year intervals.

In this study, the data collected five times, over a nine-year period up to Apr 15, 2018, were used. To apply the same air pollution dataset to the student group who conducted the survey at the same period, the questionnaire was sent to the parents through the school to be filled in and sent back within one week.

The questionnaire in the survey contained the key questions suggested by the International Society of Asthma and Allergy of Children (ISAAC) as well as various questions regarding the basic personal information (age, sex, etc.), family history of allergy, socio-economic indicators (education level of patients, family income, etc.), indoor and outdoor environmental data (smoking, distance from home to the road, odor, air pollutants, ventilation, having mold, etc.). The response to the questionnaire was computerized by a trained investigator at the Environment Health Care Center of Ulsan University Hospital.

To detect the internal inconsistency for each round of the survey, the questionnaires were rechecked for cases where the recorded response was not from among the multiple choices provided or was in contradiction to the response from another questions, and the responses of such cases were recorded again.

**Fig 3:**
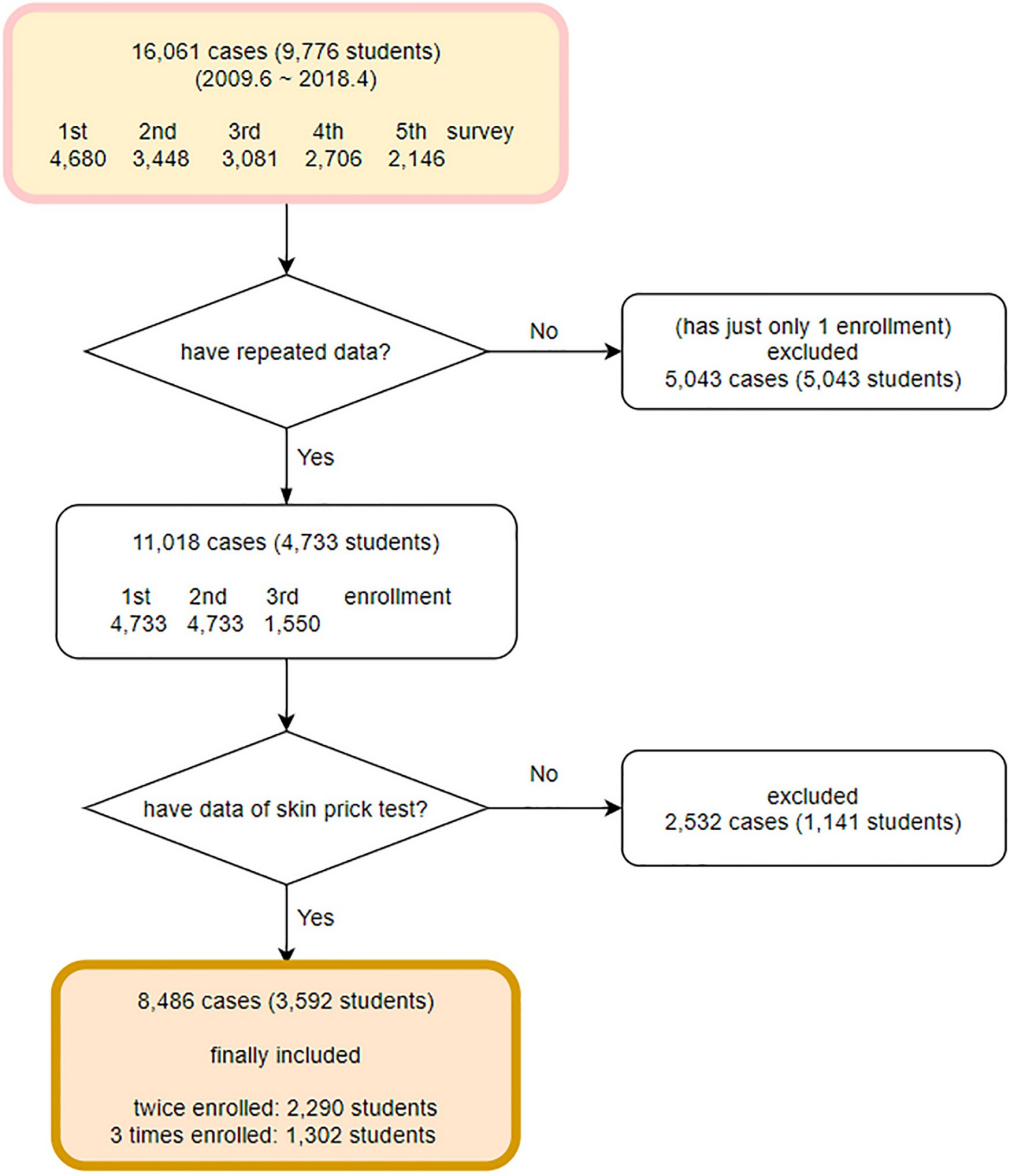
Case selection.

The students whose data of repeated measurements was available were selected, thus providing the data of 11,018 cases, while the data of 5,043 students with only a single measurement were excluded. The 11,018 cases comprised the responses of 4,733 students who participated in the survey up to two or three times. Among 4,733 students, 1,141 students who never conducted SPT were excluded in this study. After all, total 8,486 cases of 3,592 students were used in analysis. The data were categorized based on the time of survey of each student, and the result showed that 3,592 students had participated in the first and second surveys, while 1,302 students had participated up to the third survey (Fig 3) The cases in which an error was found to be revised accounted for 1.72% of all the follow-up data, including 112 cases of errors in recording and 78 cases of recording in a wrong order.

Surveys were conducted 1~3 times per student and data of 16,061 cases were collected from 9,776 students in all. The number of participating students and the response rate for the five rounds of the survey were as follows, from 1 to 5 respectively: 4,680 (83.9%), 3,448 (87.8%), 3,081 (89.3%), 2,706 (92.7%), and 2,146 (74.6%).

The general characteristics of the included and excluded students were examined (Table 2). Compared to the included students, the excluded students were from higher levels of the school year at the time of survey, and had higher age, height, and weight. This is presumed to be due to the overall participation rate, as the students who had participated in the survey during lower school years continued to participate in the higher years, while those who had already been in year 5 or 6 at the time of their first survey were excluded from the follow-up. Thus, students excluded from the follow-up showed a lower mean of the number of survey and a higher mean of the school year. In addition, the one-year diagnosis of major diseases generally showed no significant difference, except for allergic conjunctivitis.

**Table 2:** General characteristics of the excluded and included students.

	Excluded students (n = 6,184)	Included students (n = 3,592)	P-value^†^
Number of Survey *	2.38 ± 1.61	2.76 ± 1.27	< 0.001
School year *	3.72 ± 1.87	3.57 ± 1.63	< 0.001
Male:Female	1: 0.95	1: 0.97	0.305
Age *	9.18 ± 1.92	9.08 ± 1.71	0.003
Height *	138.27 ± 13.10	136.74 ± 11.79	< 0.001
Weight *	35.28 ± 13.80	33.96 ± 10.17	< 0.001
Asthma^‡^	1.8%	2.0%	0.380
Allergic rhinitis^‡^	26.8%	27.7%	0.280
Atopic dermatitis^‡^	12.4%	11.6%	0.150
Allergic conjunctivitis *^‡^	11.8%	13.0%	0.043
Food allergy^‡^	2.2%	2.6%	0.170

^†^: p-value by student’s t test or chi-squared test (categorical variables).

*: p-value < 0.05.

^‡^: history of diagnosis by the doctor in the last one year.

After explanation of the survey, parents of all participants were provided written informed consent prior to participation. This survey was approved by the Institutional Review Board of the Ulsan University Hospital (approval no. UUH 2009-09-061).

#### Skin prick test

SPT was performed as an objective indicator for determining the allergic sensitization of a student. The standard antigens used in the SPT were as follows: negative control (physiological saline), positive control (histamine), American mite, European mite, mold mite, cockroach, ragweed, Asiatic plantain, willow, mugwort, Japanese hop, Japanese alder, dog, cat, indoor mold, outdoor mold, Koji mold, Japanese white birch, oak, pine, white goosefoot, maple, shrimp, flour, milk, and egg. A student was categorized as SPT positive if he or she showed at least one positive reaction to a minimum of one antigen among the 24 standard antigens listed above.

#### Concentration of air pollutants

This study used the monthly average data from 13 air quality monitoring sites (AQMS) managed by the National Institute of Environmental Research (NIER) and the Community Multiscale Quality (CMAQ) model (version 5.0.1) [28, 29] predictions to estimate the environmental exposure of participants to air pollutants (Fig 2).

Assuming that the students resided in an area close to their respective elementary schools and spent most of their time at either school or home, the average air pollutant exposure level of the students at each school was estimated based on the data of air pollutant concentrations collected from the monitoring site close to the school.

**Fig 4:**
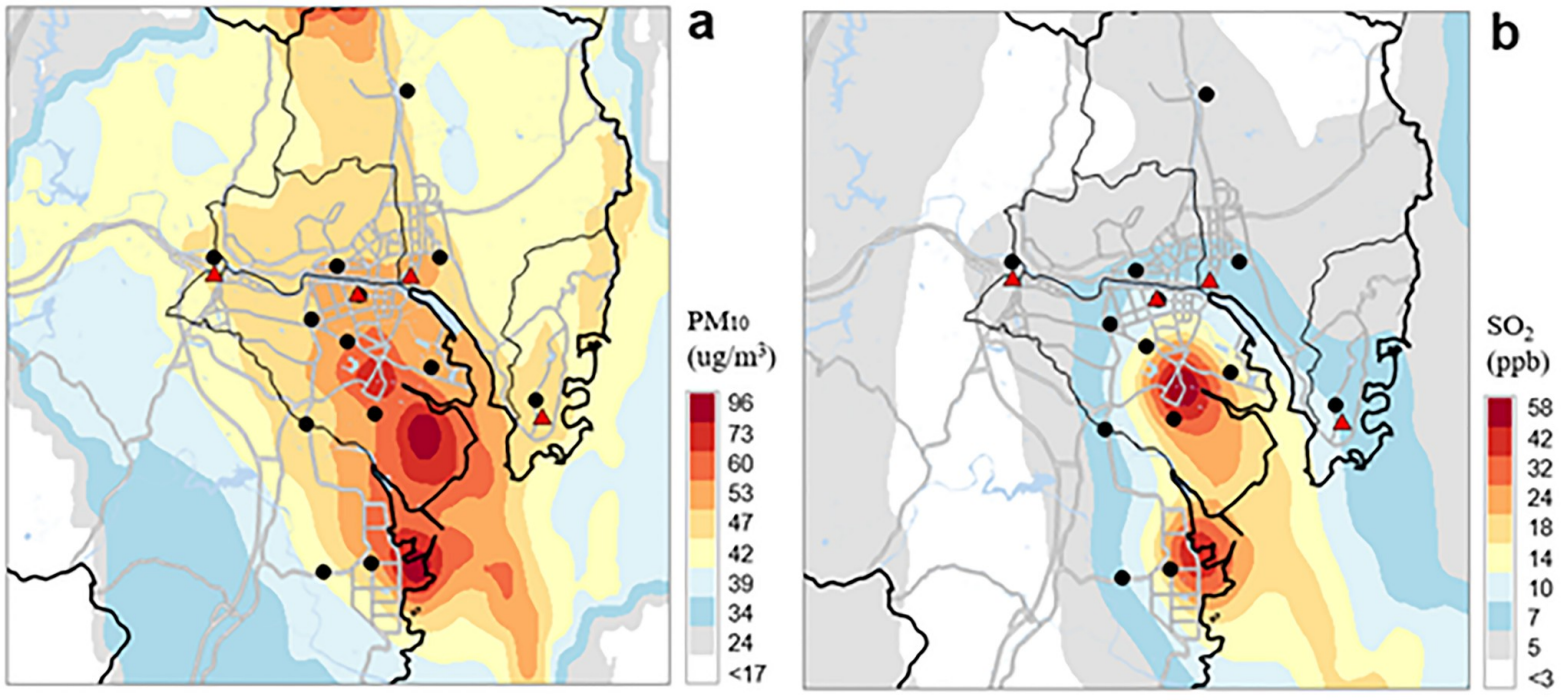
Horizontal variations of annual mean (a) PM10 and (b) SO_2_ concentrations predicted by fusing CMAQ predictions (1 km grid spacing) with monitored concentrations from air quality monitoring sites (AQMS) for 2014. Black dots and red triangles indicate AQMS and four study schools, respectively.

The CMAQ model was run for one year of 2014, in the middle of study period, in a nested mode at 27, 9, 3, and 1 km horizontal grid dimensions; The fine-scale innermost domain is the target area of the study that covers Ulsan and the surrounding area, which is the same as one used in Bang et al.(2018)’s study [30]. The detailed CMAQ-ready meteorological and emission inputs, initial and boundary conditions, and physical and chemical options used in our CMAQ modeling were described in Oh et al. (2021)’s study [31]. The hourly 1-km gridded concentrations predicted by the CMAQ model were averaged on monthly basis and then were blended with ambient monitored data from 13 air quality monitoring sites in Ulsan (AQMS) (Fig 2). This fusion process combines the inverse distance weighting (IDW) method for spatial monitored data interpolation with spatial scaling using gridded CMAQ predictions [31, 32]. For example, Fig 4 shows the fused annual mean concentrations of PM10 and SO_2_ for 2014. Adjusted concentrations by fusing monitoring data with CMAQ prediction can provide more reliable concentration information at four school areas because there can be significant in concentrations differences between school area and monitoring site. We assumed these fused gridded concentrations at school areas as exposure estimation for participants. Several previous studies have used this fusion technique to improve the CMAQ predictions for air pollution exposure estimation [31–34].

The result of the air pollutant measurements is presented in Table 3, for each round of the survey and the schools.

**Table 3:** The mean annual concentration of air pollutants of the last one year of the survey, classified by schools.

School †	Date of survey (year. month)	NO_2_ (ppb)	SO_2_ (ppb)	O_3_ (ppb)	CO (ppb)	PM10 (*μ*g/m^3^)
A	9.07	24.9	6.3	34.1	457.3	50.4
	12.03	30.3	4.5	30.1	515.9	43.5
	14.05	34.4	6.4	40.8	517.2	45.1
	16.04	27.2	4.3	42	458.3	38
	18.04	29.8	4.1	41.4	541.5	38.2
B	9.07	22.1	4.7	39.3	497.1	50.3
	11.03	23.4	5.4	37.9	485.4	51
	13.03	23.5	5.1	42.3	544.8	49.6
	15.04	23	5	41	508.5	49.8
	17.04	21.7	4.4	44.9	506.3	40.5
C	9.07	19.3	7.6	34.2	466.8	45.1
	11.03	18.9	8	36	526	45.7
	13.03	19.4	8.8	42.5	511.8	48.6
	15.04	18.9	7.6	41.6	566.5	46.4
	17.04	17.4	6.7	40.8	484.2	42.8
D	10.03	32.8	6.1	35.8	380	48.8
	12.03	23.2	6	33.7	503	45.5
	14.04	25	8.5	40.1	534.7	45.5
	16.04	22.6	7.1	40.2	495	42.8
	18.04	22.6	6.5	43	600.5	39.7

SO_2_: sulfur dioxide, NO_2_: nitrogen dioxide, O_3_, ozone, CO: carbon monoxide, PM10: particulate matter 10 μm or less in diameter.

^†^ School A: Suburban residential, B: Near industrial, C: Coastal residential, D: Central urban.

### Statistical analysis

Among the six allergic diseases, this study focused on the analysis of allergic rhinitis that showed the highest prevalence. The prevalence indicator was the question “Have you been diagnosed with allergic rhinitis by a doctor in the past 12 months?” and the response was used to define the dependent variable of one-year diagnosis of allergic rhinitis. The indicator based on the diagnosis by a doctor to prevent the interference due to the subjective assessment of allergic rhinitis, is considered as a suitable indicator to identify the corresponding prevalence.

To determine the explanatory variables within a suitable model for describing the dependent variables, logistic regression analysis was performed from the cross-sectional analysis. We performed logistic regression analysis with a backward stepwise likelihood ratio entry method, which selected the following explanatory variables: air concentration of SO_2_, air concentration of PM10, response to the SPT, sex, age, BMI, family history of allergic diseases, education level of father, history of bronchiolitis, type of house, age of house, use of a humidifier, use of an air-conditioner, ever moved to a newly built or remodeled house, and ever had a pet. Then the concentrations of NO_2_, O_3_, and CO were added to the explanatory variables to investigate the effects of air pollutant concentrations. Finally, using the repeated measurement data, the association of allergic rhinitis with explanatory variables including air pollutants was evaluated by calculation of odds ratios (ORs) using a multivariate generalized estimating equation (GEE). To check the influence of all variables suitable in the model, variables without statistical significance were excluded in GEE.

For all statistical analyses, SPSS Statistics 21.0 (SPSS Inc., Chicago, IL, USA) was used, with the significance level set to *p* <0.05.

## Results

With the variables selected via the logistic regression analysis as the explanatory variables, the GEE analysis was performed for the prevalence of allergic rhinitis using the data of one-year diagnosis, and the results are presented in Table 4.

**Table 4:** Factors related to prevalence of allergic rhinitis by last-1-year diagnosis by multivariate generalized estimating equation. (n = 3452).

Variable	OR	95% CI of OR	p-value
NO_2_ (ppb)	1.002	0.987–1.017	0.816
SO_2_ (ppb)*	1.056	1.006–1.109	0.028
O_3_ (ppb)	1.006	0.990–1.023	0.447
CO (ppb)	1.000	0.999–1.001	0.926
PM10 (*μ*g/m^3^)*	0.979	0.962–0.997	0.021
Age (year)	0.967	0.933–1.002	0.064
BMI (kg/m^2^)*	1.021	1.005–1.038	0.008
Sex (female vs. male)*	0.796	0.696–0.911	0.001
SPT (positive vs. negative)*	1.828	1.589–2.104	< 0.001
Father’s allergy history* (yes vs. no)	1.956	1.718–2.227	< 0.001
Mother’s allergy history* (yes vs. no)	2.390	2.100–2.721	< 0.001
Sibling’s allergy history* (yes vs. no)	1.508	1.323–1.720	< 0.001
History of bronchiolitis under 2 years of age* (yes vs. no)	1.338	1.126–1.591	0.001
Type of house			
Segregated house		reference	
Multi-family*	1.731	1.174–2.554	0.006
Apartment*	1.625	1.164–2.269	0.004
Age of the house			
< 1 year		reference	
1–4 years*	0.658	0.434–0.998	0.049
5–9 years*	0.568	0.381–0.847	0.006
10 years or over*	0.626	0.423–0.927	0.019
Humidifier (yes vs. no)*	1.305	1.143–1.490	< 0.001
Air-conditioner (yes vs. no)*	1.486	1.212–1.821	< 0.001
Moved to new home (yes vs. no)	0.981	0.863–1.114	0.763
Pets (ever vs. never)*	0.842	0.726–0.975	0.022

The results indicated a significant positive correlation between the prevalence of allergic rhinitis and the concentration of SO_2_ among the analyzed air pollutants (OR 1.056), whereas a significant inverse correlation with the concentration of PM10 (OR 0.979) was found. No correlation was found for other air pollutants. A positive result of the SPT was significantly correlated with allergic rhinitis (OR 1.828). The risk of allergic rhinitis was significantly high if a parent or sibling showed a history of allergic disease, and a history of allergic disease in the mother was shown to be the most influential variable (OR 2.390). The risk of allergic rhinitis was high in the student who lived in a house built within the past year, and it was higher in those who lived in multi-family building or apartment than in a segregated house. The risk was significantly higher in male students than in female students, and in the students who had a history of bronchiolitis at less than two years of age. The risk of allergic rhinitis was also higher in the student with higher BMI. The use of a humidifier or air conditioner was also found to be associated with an increased risk of allergic rhinitis. The history of living with a pet was correlated with a lower risk of allergic rhinitis.

## Discussion

The health hazards caused by exposure to air pollution are particularly concerning in children, as their immune system and lung development are not adequately mature against the air pollutants; Hence, the level of hazard is much higher than in adults [35]. In addition, as children spend considerably more time outdoors than adults do, they are more susceptible to air pollution [36].

This study focused on determining the association of allergic rhinitis with air pollutants, among the various potential factors. The results showed a significant association between the prevalence of allergic rhinitis and the concentrations of SO_2_ and PM10, among the air pollutants investigated.

This study showed that SO_2_ concentration was associated with an increased risk of allergic rhinitis with an OR of 1.056 (1.006–1.109), a significant finding that confirmed a partial influence of the industrial complex on the prevalence of allergic rhinitis. SO_2_ is produced from organic decomposition or volcanic eruption in nature, while it is mostly considered to be a combustion byproduct of organic fuels such as petroleum in industrial areas, and serves as an indicator of the level of urbanization or industrialization [37].

Industrial facilities, including factories, a power plant, and oil refineries, are the main sources of SOx emissions in Ulsan (Fig 2), and emissions from the petrochemical complex are predominant throughout the year [38]. Additionally, ship transportation in the coastal area near a shipbuilding plant is a significant source of SOx emission that can influence ambient SO_2_ concentrations at most regions near the coast. This explains the higher atmospheric SO_2_ concentrations at Schools C and D than at School B under certain wind conditions, although School B is closest to the industrial complex (an automobile plant). The predominantly westerly winds in Ulsan throughout the year explain why the SO_2_ concentration at school C (the western-most school) was highest among the four schools (Table 3, Fig 2). Differences in SO_2_ concentrations at the four Schools can also be affected by uncertainties in SOx emission input from CMAQ modeling and differences in the estimated periods of SO_2_ exposure.

In previous studies on the relationship between SO_2_ and the prevalence of allergic rhinitis, OR in the range of 1.14–1.43 was reported in a cross-sectional study conducted by Hwang et al. among 32,143 elementary and middle school students in Taiwan [19], while Kim et al. conducted a cross-sectional study among 4,545 elementary school students in Korea and reported an OR of 1.30 [18]. In the study by Kim et al. [18], the geographic locations of the elementary schools were categorized into hometown zone, traffic related zone, and complex source zone. In the hometown and traffic related zones, no significant correlation with SO_2_ was found, while in the regions close to the industrial complex, a significant correlation between SO_2_ and allergic rhinitis was observed. The measured values of SO_2_ were reported to be 4 ppb for the hometown and traffic related zones and 5–7 ppb for the complex source zone. Based on the findings in the present study wherein the SO_2_ concentration was in the range of 4.1–8.5 ppb, we can consider the possibility that an environment with SO_2_ concentration above a certain level leads to allergic rhinitis in school-age children. In a meta-analysis of nine studies that investigated the relationship between allergic rhinitis and SO_2_ concentration during the period Jan 2000 –Feb 2018, the finding from the data of 46,392 children showed an OR of 1.085 [39]. Among the nine reviewed studies including four cohort studies, no study reported a significant correlation between the prevalence of allergic rhinitis and SO_2_ [40–43]. The statistically significant correlation between SO_2_ and allergic rhinitis in the present study is thus significant in confirming the findings of the previous cross-sectional studies as well as in detecting the rare statistical significance in the follow-up data.

Few previous studies investigated the relationship between allergic rhinitis and the level of PM10 in elementary school students. One study reported a significant relationship (OR = 1.14, 95% CI = 1.02–1.27) [20]. However, a meta-analysis of studies that used the ISAAC questionnaire reported an OR of about 1, with little influence and inconsistent directionality among studies [44]. A prospective cohort study of 3,049 schoolchildren showed no significant correlation between PM10 and asthma [45]. Another study showed that seasonal allergic rhinitis was not associated with PM10 but was associated with temperature [46].

Kang et al. investigated changes in allergic symptoms in 108 patients with allergies and 47 healthy subjects by comparing their 120-day symptom scores and found that none of the allergic symptoms correlated with daily PM10 changes. However, their data indicated that allergic symptoms were significantly aggravated by an increase in time outdoors and that temperature had a significant effect in both groups [47]. They concluded that their results were similar to those of a previous ISAAC study in Japan [45, 47]. There are few longitudinal studies of the link between PM10 and allergic rhinitis, and the results of Kang et al. are similar to those of the present study.

PM10 can act as carriers of allergen, aggravating allergic reactions, and smaller particles can enter the respiratory tract, breaching the mucosa [39, 48]. However, since it is difficult to identify the source or mechanism of the release of PM10, the interpretation of the result is challenging because PM10 considered the size of the dust particle rather than the characteristics of the components [39]. Few previous studies investigated the relationship between the composition of PMs and allergy. A longitudinal study found that total PM2.5 was not significantly associated with any asthmatic symptom or medication use. However, a detailed analysis of the composition of PM2.5 found that PM2.5 mass concentration coming from motor vehicles or road dust was consistently associated with increased likelihood of respiratory symptoms and inhaler use [49]. Another study of the effect of PM sources in children showed that oil had significant negative association with the symptom of wheezing [50].

The present study did not find the association between NO_2_ concentration with allergic rhinitis. The association between NO_2_, the biomarker of traffic-related air pollutants, with allergic rhinitis has been widely reported in adults and children in Asia and Europe [19, 51–58]. However, the association between NO_2_ and allergic rhinitis has not always been consistent. Ambient SO_2_ concentration was positively correlated with the prevalence of allergic rhinitis, but there was no correlation with other air pollutants such as PM10 and NO_2_ in Chinese adults [59]. De Marco et al. showed that NO_2_ was positively associated with allergic rhinitis in the Mediterranean region but not in the subcontinental region in Italy [60]. They argued that that result implies that the climate interacts with outdoor air pollutants. A Korean study on elementary school children showed a significant OR of 1.34 (1.17–1.53) in complex areas around the factory and an OR of 0.71 (0.54–0.91) in areas with heavy traffic [20]. Further prospective studies are needed.

For the analyses of other environmental factors, the risk of allergic rhinitis was significantly higher in the students living in a house built within the past year. This might be attributed to the building syndrome caused by the construction materials of a newly built house [61]. For example, a study of newly built houses in Japan reported that some volatile organic compounds (VOCs) and formaldehyde had significant dose-dependent relationships with nonspecific subjective symptoms that resembled “sick building syndrome” [62]. A study in Korea reported that the inner walls of many homes are covered with materials that emit air pollutants, including VOCs and formaldehyde, and this increased the risk for atopic dermatitis in children [63]. Huss-Marp et al. showed that exposure to VOCs damaged the epidermal barrier and worsened atopic dermatitis at concentrations commonly found in indoor environments [64].

The risk of allergic rhinitis in this study was higher in students staying in a multi-family building or in an apartment than in a segregated house. This might be attributed to the high humidity and moldiness observed in a multi-family building [65]. It could also be conjectured that living in a multi-family building increases the chances of exposure to cigarette smoke if there is a smoker among the neighbors.

Some studies found high risk of building syndrome in a newly built house, multi-family building, and public housing. These studies suggested that the age of each building, the ownership, and the characteristics of the residents should be examined in addition to living in a multi-family building [66–68]. Although the questionnaire in this study did not include any item on the ownership, the results could be interpreted in line with the previous studies. We also investigated the effect of the type of heating fuel used in each home, but this factor was not significant in the logistic regression, so was not included in the final analysis.

The present study found a significant correlation between the use of a humidifier or an air conditioner with the risk of allergic rhinitis, although history of living with a pet had a significantly lower risk. This may be because parents with a child who has allergic rhinitis may eschew pet ownership, but use an air conditioner or air purifier more frequently.

The interpretation becomes slightly more complicated in the case of use of humidifier. A study reported bacterial growth inside the humidifier [69], while another reported the problem of bacterial or fungal growth caused by the moisture on the inner wall, on continuous use of the humidifier [70]. However, another large-scale cohort study among young children found no association between the use of humidifier and either house mites or fungi [71]. For the correlation found in this study between the use of humidifier and the prevalence of allergic rhinitis, the causality cannot be precisely defined based solely on the results.

Among the non-environmental factors shown to have a correlation with the prevalence of allergic rhinitis, the one with the highest risk was the history of allergy in a parent or sibling. Among other variables, a significantly higher risk was associated with SPT positivity, history of bronchiolitis, female students than in male students, and BMI. For the family history of allergy, where the temporal relationship is clear and the conditions cannot be arbitrarily modified unlike in the case of the use of humidifier or air conditioner, the results might be interpreted as the cause-effect relationship. The factor that showed the highest OR was the history of allergy in the mother. The fact that the first and second factors with the highest OR were the history of allergy in the mother and father clearly indicates the presence of a genetic factor in allergic rhinitis. Numerous previous studies have reported the correlation between the history of allergy in a parent and the probability of an allergy in the child [72–76]. A recent study also showed a significant increase in the risk of allergic diseases including asthma, allergic rhinitis, and atopic dermatitis, if a parent had a history of allergy [77]. Research on the association between family history and allergic diseases has been widely conducted, and recent studies have shown the association of specific genetic polymorphism with the susceptibility to allergic rhinitis [78, 79].

The findings in this study also confirmed the high correlation between the SPT positivity and the prevalence of allergic rhinitis [80]. Though SPT cannot predict the severity of illness by stratifying the size of the skin-prick test result, SPT can be applied to support the diagnosis of allergic rhinitis [81]. Jung et al. suggested that SPT is the recommended first choice than serum IgE level measurement for detecting allergy to house-dust mites in patients <30 years old [82].

A history of bronchiolitis below the age of two years showed a higher risk of allergic rhinitis, which might be interpreted in two different ways based on the previous studies. First, there is a report on the correlation between an increased risk of asthma or allergic rhinitis, and the use of antibiotics during the early days after birth that alters the normal flora in the gastro-intestinal tract [83], based on which the correlation between the history of bronchiolitis and the prevalence of allergic rhinitis in this study can be explained. As another plausible explanation, a study reported an incidence of allergic disease caused by glutathione deficiency due to the reduced protective function of antioxidants in the respiratory mucosa because of the use of paracetamol (the most well-known product name is Tylenol^®^), a drug most commonly used to treat inflammation and fever [77, 84]. Ever since Aspirin^®^ was reported to cause Reye’s syndrome, the use of paracetamol is highly likely as there are limited options for an antipyretic that can be used to reduce the fever in infant, which thus lends support for the finding in this study.

This study had several limitations. First, we were unable to evaluate the exposure level of each individual student. Instead, we estimated the air pollutant exposure at each school based on average air pollutant concentrations at nearby monitoring sites. We believe these measurements meaningful because all students resided close to their schools, spent most of their time at school or home, and we provided more reliable adjusted concentrations at each school by fusing monitoring data with CMAQ predictions. Second, most values were based on questionnaire responses rather than objective measurements, so recall bias or misclassification bias is possible. Third, we used mean annual concentration of air pollutants of the last one year of the survey, but not changes of concentrations, seasonal changes, or short-term concentrations in the analysed period. However, uncertainties may be decreased because we used a diagnosis with allergic rhinitis by a doctor in the past 12 months as outcome variable. Finally, we did not include school-related factors, such as the age of the building, humidity and moldiness, building materials, and heating systems that vary among schools, because of the strong multicollinearity between measured air pollution and school-related variables.

The strength of the present study was that we examined a large population over a long period of time and there was little variation in the subjects, as the study investigated all students at the elementary schools. Hence, the association between air pollutants and the prevalence of allergic rhinitis could be verified.

In conclusion, the concentrations of SO_2_, which is indicator of the degree of industrialization, was related to the prevalence of allergic rhinitis. Among all the risk factors, history of allergic disease in the parents was the most important factor, and the study reconfirmed the results of the previous studies. Further research with the measurement of indoor and outdoor factors at each home and the schools is needed.

## Supporting information

S1 File (SAV)

S2 File (SPS)

S3 File (DOCX)
