# Supplementary material for: Allergic rhinitis is associated with atmospheric SO2: Follow-up study of children from elementary schools in Ulsan, Korea
Source: PLoS One. 2021 Mar 18;16(3):e0248624. doi: 10.1371/journal.pone.0248624 (PMC7971526; doi:10.1371/journal.pone.0248624)

Questionnaire (part)

Gender male/female

Who are you? Child’s

1. Father 2. Mother 3. Grandparents

4. Relative 5. ETC ( )

Answering date: Year month day

School/grade/class/number / / /

Birthdate

Name

Height/weight: cm kg

Home address city gu road (building No.)

floor (house No.)

Has your child ever been diagnosed for allergic rhinitis **in the past 12 months**? no/yes

History of allergic diseases of family members.

Has your child's father ever been diagnosed or treated for the following diseases?

1. Asthma no/yes
2. Allergic rhinitis no/yes
3. Atopic dermatitis no/yes
4. Allergic conjunctivitis no/yes
5. Other allergic diseases (urticaria, allergy on metal, bug, drug, rubber) no/yes

Has your child's mother ever been diagnosed or treated for the following diseases?

1. Asthma no/yes
2. Allergic rhinitis no/yes
3. Atopic dermatitis no/yes
4. Allergic conjunctivitis no/yes
5. Other allergic diseases (urticaria, allergy on metal, bug, drug, rubber) no/yes

Has your child's brother or sister ever been diagnosed or treated for the following diseases?

1. Asthma no/yes
2. Allergic rhinitis no/yes
3. Atopic dermatitis no/yes
4. Allergic conjunctivitis no/yes
5. Other allergic diseases (urticaria, allergy on metal, bug, drug, rubber) no/yes

Has your child ever been diagnosed with bronchiolitis by a doctor within 2 years (24 months) of birth? no/yes

What type of house do you live in?

1. Segregated house
2. Multi-family building
3. Apartment

How long has your house been built?

1. Less than 1 year
2. 1 year ~ less than 5 years
3. 5 year ~ less than 10 years
4. 10 years or over

Please v mark what you use in your home.

1. Humidifier no/yes
2. Air-conditioner no/yes

Has your child ever moved to a new or renovated home? no/yes

Has your child ever had pets at home? no/yes


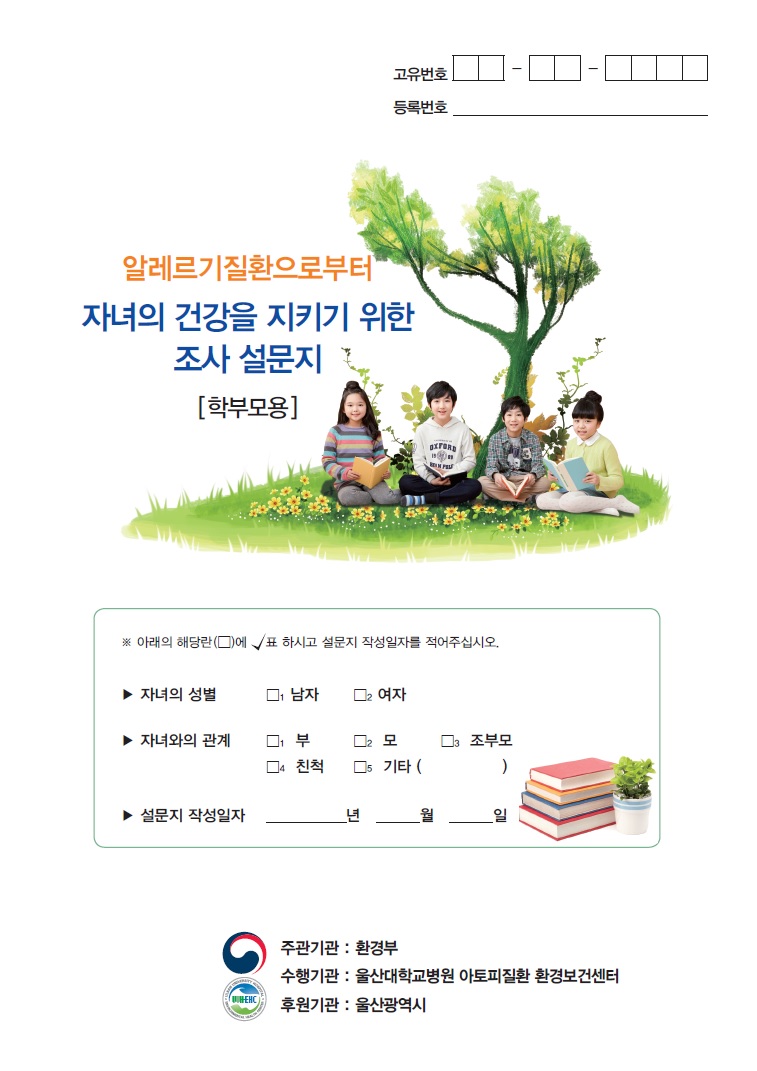
Korean full version


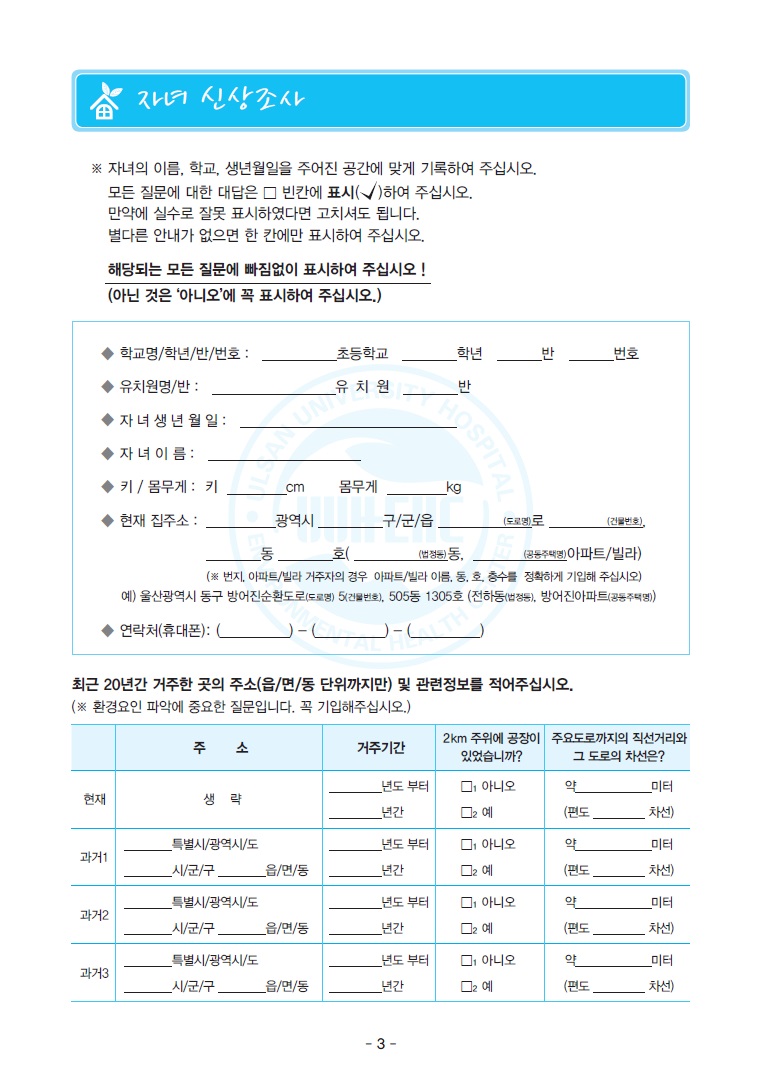


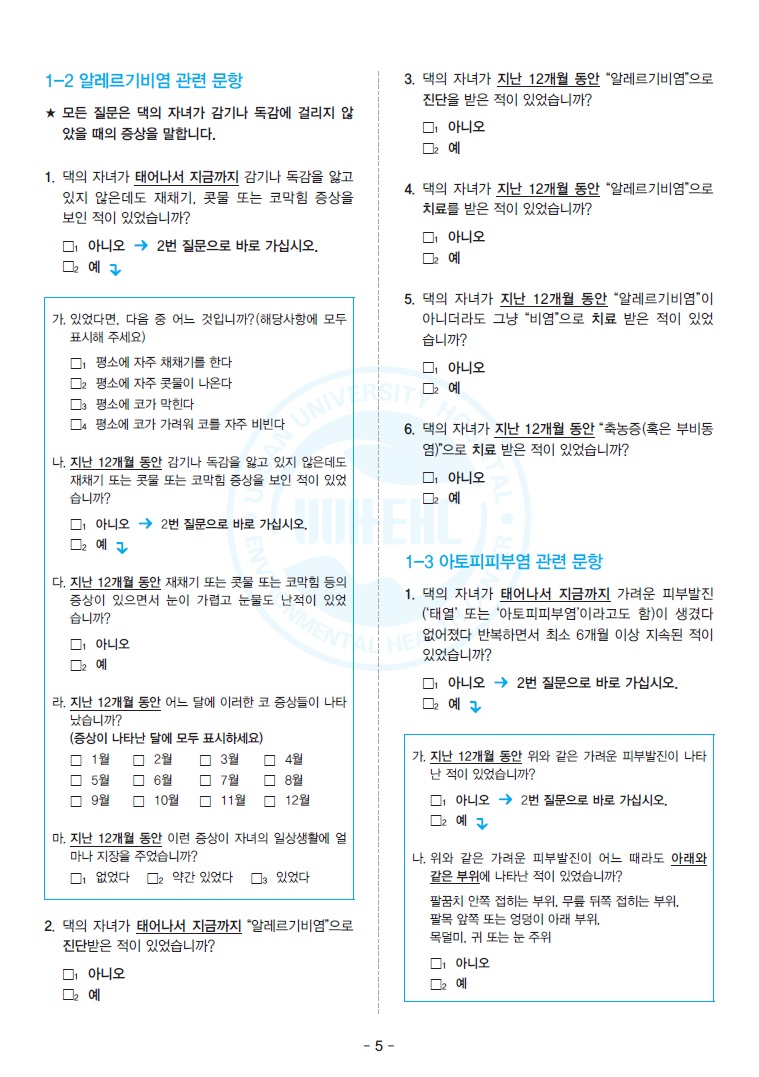

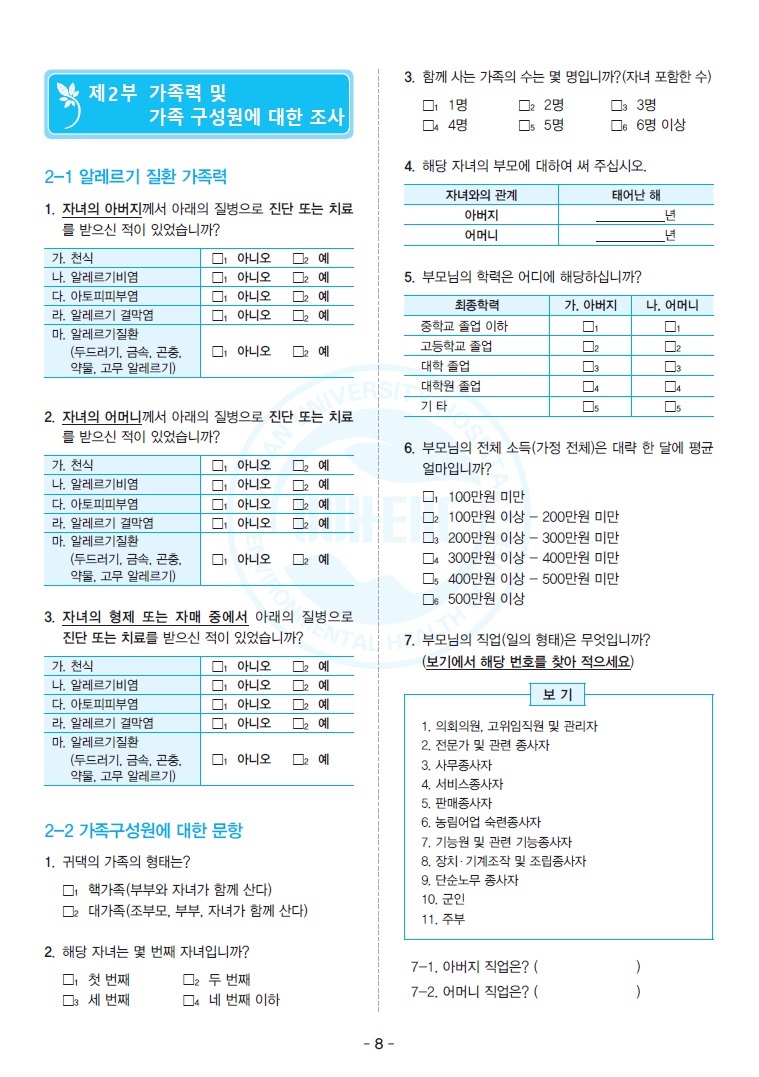

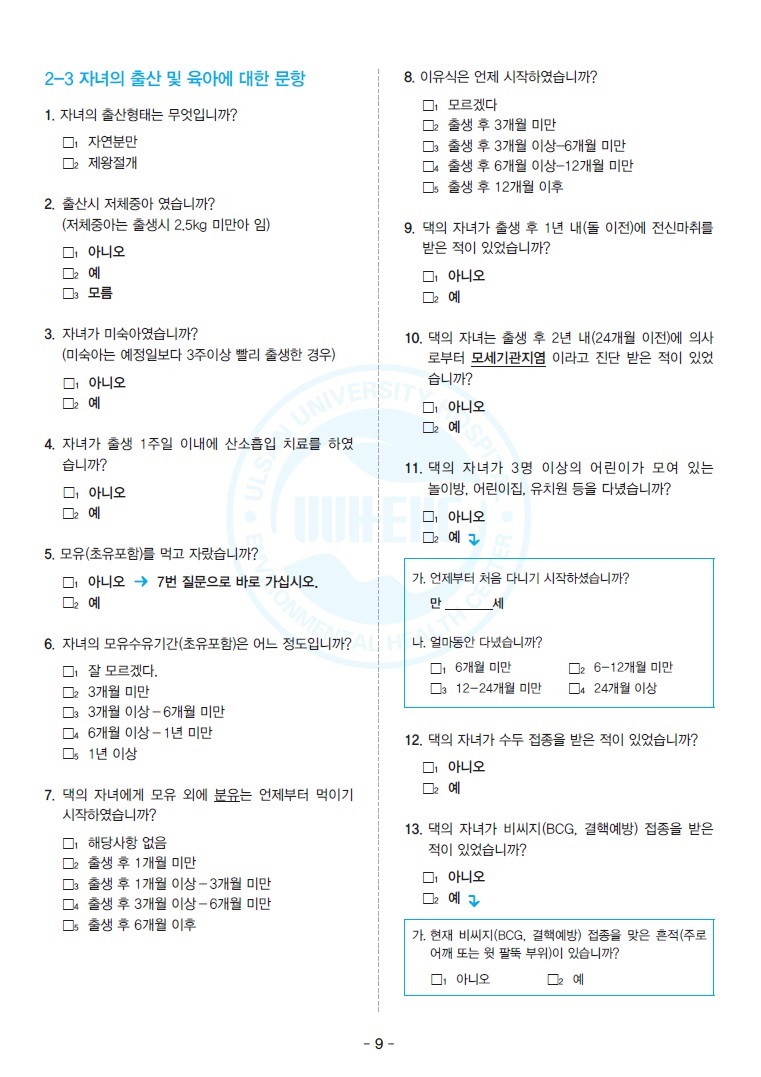

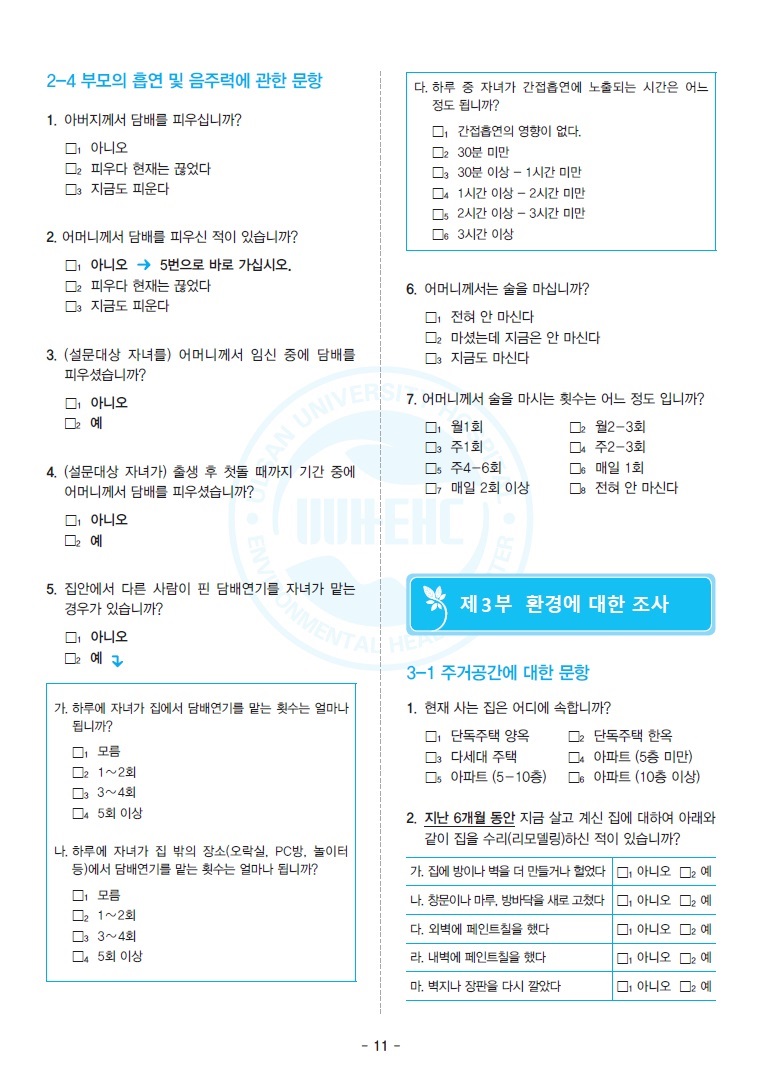

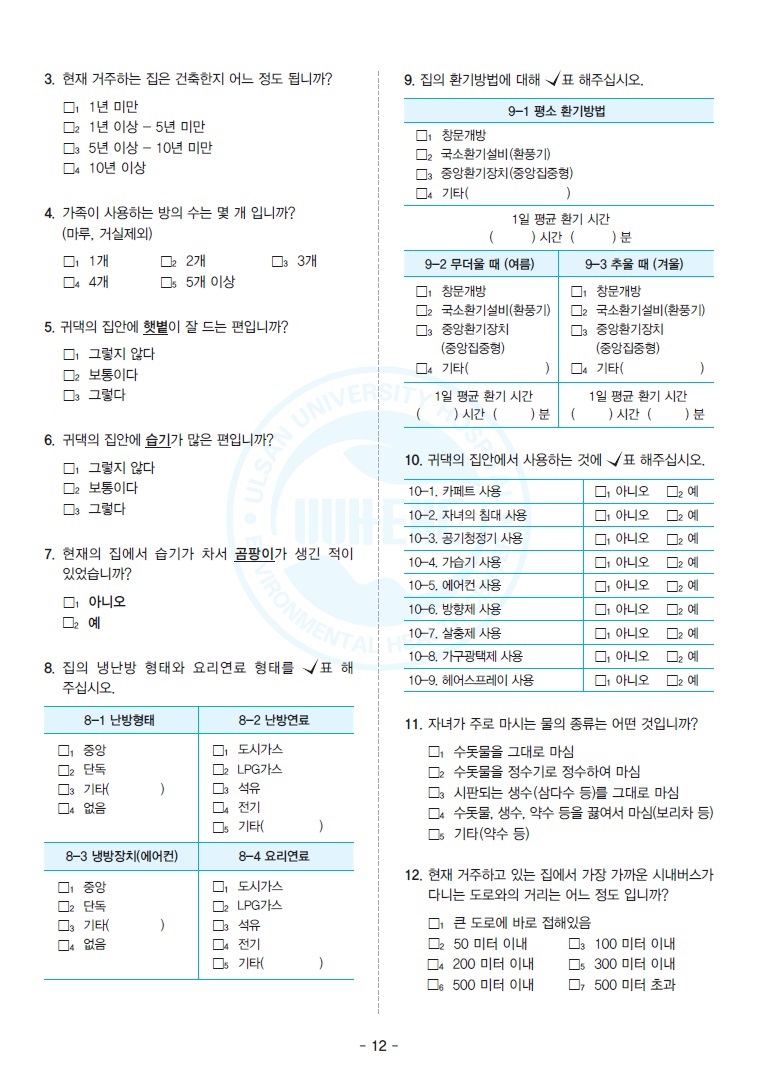

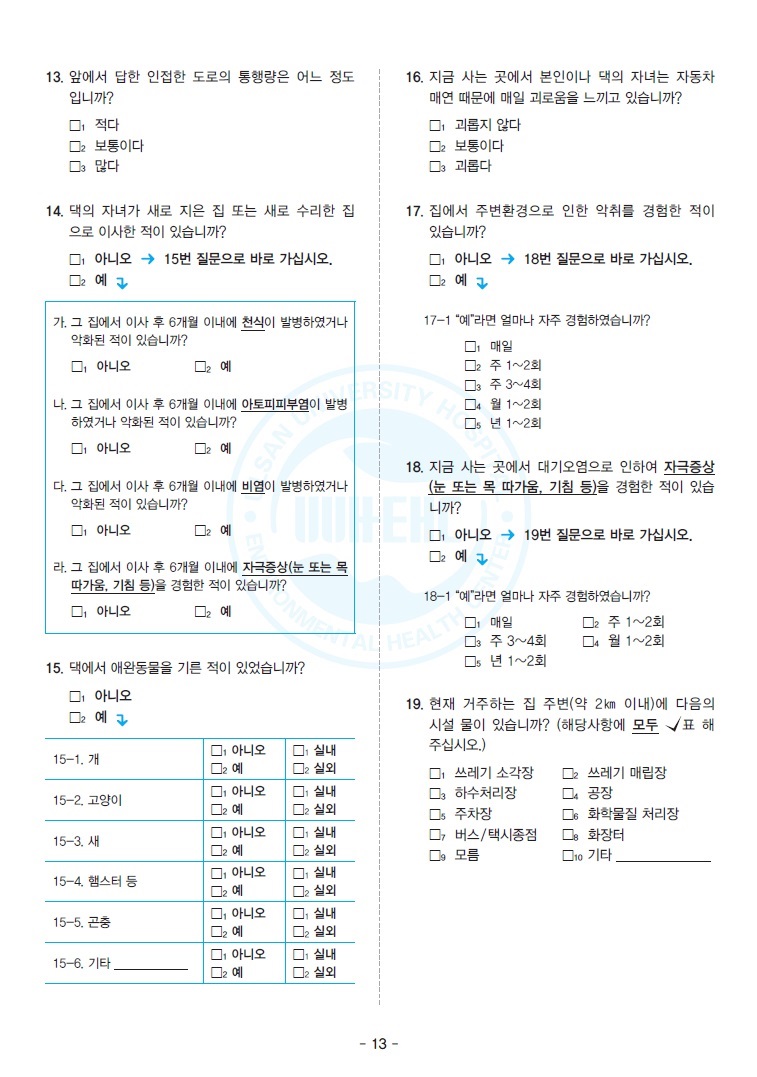

Supplement: S3 File — (DOCX) [file pone.0248624.s003.docx]
